# Bubble Nucleation and Growth in a Force-Driven Flowing Liquid Film Under Controlled Pressure by Molecular Dynamics Simulation

**DOI:** 10.3390/ma19061154

**Published:** 2026-03-16

**Authors:** Ziqi Li, Ziqi Cai, Zhengming Gao

**Affiliations:** 1 State Key Laboratory of Chemical Resource Engineering, Beijing University of Chemical Technology, Beijing 100029, China; 2 School of Chemical Engineering, Beijing University of Chemical Technology, Beijing 100029, China

**Keywords:** bubble nucleation, molecular dynamics simulation, liquid flow, applied force, pressure control, bubble growth

## Abstract

Bubble nucleation in flowing liquid films is a common interfacial phenomenon affecting the heat and mass transfer at the solid–liquid interfaces in many thermal and functional material production processes, yet realizing its molecular-scale mechanisms under coupled flow, pressure, and heating conditions is important. In this study, molecular dynamics simulations are performed to investigate the bubble nucleation and growth in a liquid argon film on a heated platinum substrate under controlled pressure, with liquid flow driven by an applied body force. Bubble evolution is analyzed by the nucleation time, critical nucleation volume, bubble volume variation, and migration of the bubble’s center of mass. The results show that system pressure and substrate temperature dominantly regulate the nucleation: increasing pressure delays nucleation, whereas increasing substrate temperature accelerates it. Under a fixed system pressure and substrate temperature, liquid flow exhibits a non-monotonic influence. The applied forces from $4.0\times 10^{-7}\;eV/Å$ to $1.0\times 10^{-6}\;eV/Å$ gradually promote the nucleation and enhance the bubble growth by facilitating near-substrate heat transfer and density fluctuations, while the forces from $1.0\times 10^{-6}\;eV/Å$ to $1.4\times 10^{-6}\;eV/Å$ suppress nucleation and do not further promote the growth due to the intensified shear and interfacial instability. These findings provide molecular-level insight into the coupled thermodynamic and kinetic effects of pressure, temperature, and flow on bubble nucleation and growth at material interfaces, offering guidance for the design and operation of heat-transfer and functional materials processes.

## 1. Introduction

Bubble nucleation is a fundamental interfacial phenomenon that widely occurs in both natural environments and materials-based engineering systems. In boiling heat transfer, bubble nucleation and growth at solid–liquid interfaces play a decisive role in determining heat transfer efficiency and critical heat flux, forming the physical basis of high-performance thermal materials and devices such as heat exchangers and electronic cooling substrates [1,2]. In polymer processing and devolatilization [3,4], bubble formation directly governs melt morphology, porosity evolution, and final material properties, making nucleation behavior a key factor in materials processing and quality control. Moreover, controlled bubble nucleation has been increasingly exploited in nanomaterial fabrication and micro/nanoscale functional materials, where it enables the construction of porous structures, nanostructures, and stimuli-responsive material systems [5,6,7]. Therefore, a comprehensive understanding of bubble nucleation mechanisms, particularly at material interfaces and under nanoscale confinement, is essential for advancing materials design, processing optimization, and functional performance.

Early understanding of bubble nucleation was primarily established on the basis of classical nucleation theory (CNT). Originally proposed by Volmer [8], Farkas [9], and Becker [10], CNT describes nucleation as a thermally activated process governed by a free-energy barrier. The theory provides the thermodynamic framework for phase-transition phenomena and offers qualitative insight into the roles of temperature, pressure, and interfacial energy in nucleation processes. Bubble nucleation is inherently a microscopic process, with critical nuclei typically having characteristic dimensions of only a few nanometers. With advances in experimental techniques, particularly high-resolution characterization methods such as atomic force microscopy [11,12,13] and other precision measurement approaches [14,15], it has become possible to observe nanoscale bubbles and interfacial structures on solid substrates. However, their spatial and temporal resolution, as well as their degree of controllability, remain limited, making it challenging to directly probe the earliest stages of nucleation. Therefore, more microscopic approaches are necessary to achieve a deeper understanding of the microscopic dynamics and underlying physical mechanisms governing bubble nucleation.

Molecular dynamics (MD) simulation is a powerful tool in investigating bubble nucleation under such conditions, as it enables direct monitoring of molecular motions and energy evolution during the formation, growth, and collapse. In this manner, MD simulation bridges classical theoretical descriptions and experimental observations at the molecular scale. Consequently, MD has been widely applied to study bubble nucleation and boiling phenomena in fluid–solid systems described by the Lennard–Jones potential. Existing MD studies have examined a variety of factors influencing nucleation behavior, including the surface nanostructures [16,17,18,19,20,21], surface wettability [22,23,24,25,26,27], liquid film thickness [28,29,30,31], substrate temperature [32,33,34,35,36,37,38], and the system pressure [39,40,41,42].

Among the various factors mentioned above, increasing the substrate temperature can promote bubble nucleation by enhancing the energy transfer across the solid–liquid interface, thereby resulting in an earlier onset of nucleation and accelerated bubble growth [32,33,34,35,36,37,38]. In addition, some molecular dynamics studies have employed pressure-control methods to investigate the bubble nucleation behavior under the constant-pressure constraints and demonstrated that system pressure can influence the onset of nucleation as well as the subsequent growth behaviors [39,40,41,42]. Although these studies have explored the effects of substrate temperature and pressure on bubble nucleation behavior, they have mainly focused on systems in which the liquid remains quiescent, whereas in many practical situations, bubble nucleation occurs in flowing liquids.

In recent years, some works have revealed how the bubble nucleation is performed under different flow conditions. One category focuses on the capillary-driven or confined flow systems in nanochannels, where liquid motion is primarily governed by the capillary forces and geometric confinement rather than the externally imposed driving. Molecular dynamics studies showed that, in flowing liquids, bubble nucleation behavior is strongly influenced by the surface roughness [43] and wall wettability [44,45]. These factors jointly regulate the spatial distribution of nucleation sites and further affect bubble formation and subsequent evolution. By contrast, another category explicitly introduces the liquid flow in molecular dynamics simulation by applying an external driving force as an independently controlled parameter. Miao et al. [46] employed molecular dynamics simulation to investigate the nanoscale flow boiling on a flat solid surface under externally driven flow, and the results showed that the boiling onset time increases with increasing driving force. Miao et al. [47] also examined the influence of nanostructured surfaces on flow boiling, shifting the research focus from external forcing to surface-structure-mediated regulation mechanisms. Their findings demonstrated that nanostructured surfaces can effectively delay the heat transfer deterioration caused by the formation of a vapor film covering the solid wall in a liquid flow system. In addition, Yin et al. [48] reported that, compared with pool boiling, driven liquid flow is more effective in enhancing the heat transfer under flow boiling conditions.

These studies indicate that the driven liquid flow can influence bubble nucleation and growth behavior. However, the underlying mechanisms by which liquid flow affects the temporal evolution of nucleation and the subsequent bubble growth at the molecular level remain underexplored. In this work, molecular dynamics simulation is employed to investigate the bubble nucleation and growth in a flowing liquid film driven by an applied body force on a solid surface under controlled temperature and pressure conditions. The bubble evolution process is described by the nucleation time and critical nucleation volume, and analyzed through the time evolution of bubble volume and the position of the mass centroid. Through these analyses, the present study explores how liquid flow, in conjunction with the temperature and pressure, affects bubble nucleation and growth at the molecular level, contributing to a further understanding of the underlying physical processes.

## 2. Simulation System and Method

### 2.1. Simulation System

**Figure 1:**
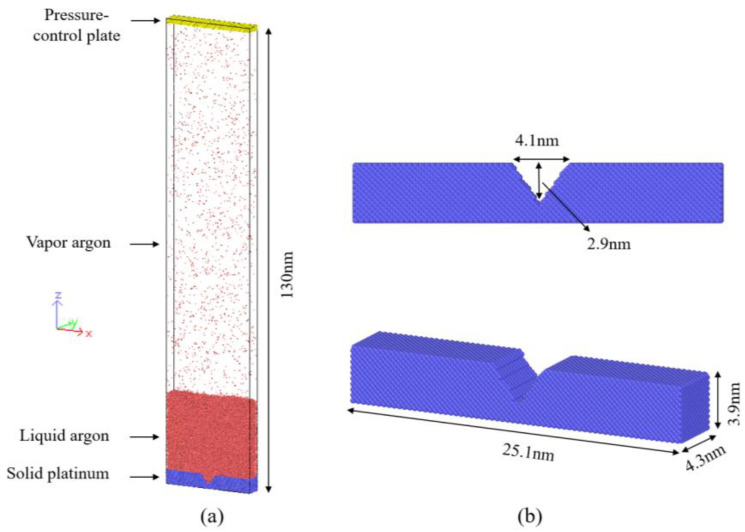
(**a**) Schematic of the initial simulation system: solid platinum substrate (blue) at the bottom, liquid argon layer (red) in the middle, and Pt pressure-control plate (yellow) at the top; (**b**) Structural configuration and geometric parameters of the solid platinum substrate.

In this study, molecular dynamics (MD) simulation is employed to investigate bubble nucleation in an argon liquid film supported on a solid substrate with cavity structures. As shown in Figure 1a, the simulation system contains only two types of atoms: argon (Ar) atoms representing the fluid phase, and platinum (Pt) atoms forming both the heating substrate and the pressure-control plate. Argon, treated as an inert monoatomic fluid, is used to describe evaporation, interfacial evolution, and bubble formation within the heated film. Platinum is selected as the solid owing to its high melting point and excellent thermal conductivity, providing a stable and controllable heat input to the liquid film. Well-established Ar–Ar and Ar–Pt interaction parameters reported in the literature are employed to model the energy transfer across the solid–liquid interface [49]. Therefore, the system is composed of three main regions: a solid platinum substrate located at the bottom (blue), a liquid argon film positioned above the substrate (red), and a platinum pressure-control plate placed at the top of the domain (yellow).

Figure 1 also shows the simulation configuration. The computational domain is a rectangular box measuring 25.1 nm in the *x* direction, 4.3 nm in the *y* direction, and 130 nm in the *z* direction. The relatively small size of the system along the *y* direction reduces the total atom count and computational cost while suppressing the formation of multiple bubbles, allowing clearer observation of individual nucleation events. In contrast, the elongated z dimension provides sufficient space for vapor accumulation, ensuring reliable evaluation of gas-phase pressure, density, and temperature. Periodic boundary conditions are applied in the x and y directions [50], while a shrink-wrapped boundary condition is used in the z direction to dynamically adjust the box size to enclose all particles and facilitate pressure regulation during phase change [51].

As illustrated in Figure 1b, the platinum substrate has a total thickness of 3.9 nm and incorporates a V-shaped groove with a depth of 2.9 nm and a width of 4.1 nm. The substrate is divided into three functional layers along the *z* direction: the bottom two atomic layers are fixed to maintain structural integrity and prevent atomic interpenetration or deformation during the heating process, the next two layers are coupled to a Langevin thermostat to provide thermal input, and the upper portion serves as a conductive layer transferring heat to the liquid argon film. The V-shaped groove promotes preferential bubble nucleation at predefined locations, enhancing the controllability and reproducibility of the simulations, as reported in previous MD studies [52,53,54].

As a result, in the simulation domain, the bottom substrate consists of 27,731 platinum atoms arranged in a face-centered cubic (FCC (111)) lattice, while 50,000 argon atoms are initially deposited on the substrate to form the liquid layer. The pressure-control plate is constructed from 9856 platinum atoms. During the equilibration stage, a fraction of the argon atoms near the free surface evaporates into the upper region, forming a vapor phase, as shown by the red particles above the liquid body in Figure 1a.

### 2.2. Simulation Method

In the present work, the interactions between Ar–Ar, Pt–Pt, and Ar–Pt atom pairs are described using the conventional 12–6 Lennard–Jones (LJ) potential, which retains the simplicity of the model while enabling the study of fundamental nucleation and growth mechanisms in this system. Lennard–Jones (LJ) potential is calculated by [55]

(1) $$\Phi \left(r_{ij}\right)=4\varepsilon \left[{\left(\frac{\sigma }{r_{ij}}\right)}^{12}-{\left(\frac{\sigma }{r_{ij}}\right)}^{6}\right],r_{ij}<r_{c},$$

where $r_{ij}$ is the distance between two particles, ε is the depth of the potential energy well, and σ is the distance at which the inter-particle potential becomes zero, representing the finite size of the particles. Considering the balance between computational accuracy and cost, the cutoff distance $r_{c}$ is set to 1.2 nm in this study [56,57].

The Lennard–Jones parameters for cross interactions between argon and platinum atoms are determined using the Lorentz–Berthelot mixing rules [58], which are given by

(2) $$\sigma_{Ar-Pt}=\frac{\sigma_{Ar-Ar}+\sigma_{Pt-Pt}}{2},$$

(3) $$\mathrm{and}\;\varepsilon_{Ar-Pt}=\sqrt{\varepsilon_{Ar-Ar\cdot }\varepsilon_{Pt-Pt}},$$

The Lennard–Jones interaction parameters between argon and platinum atoms are summarized in Table 1. To establish a stable, center-symmetric Poiseuille flow, the parameters for the pressure-control plate are set equal to those of the substrate, ensuring uniform wettability and avoiding artificial asymmetry in the velocity profile. Bubble nucleation and growth occur near the substrate and remain far from the pressure-control plate, which serves solely as a pressure boundary without contributing thermal energy, thus having a negligible effect on bubble dynamics.

The Lennard–Jones potential is a prototypical model for describing interactions between simple spherical molecules, with key advantages including clear physical interpretation, computational efficiency, and broad applicability, making it a fundamental tool in molecular simulation [59,60,61]. However, its limitations lie in its inability to capture complex molecular structures, polarity, many-body interactions, and quantum effects, and therefore extensions, parameter refinements, or coupling with more advanced simulation methods are often required for complex systems [62,63,64].

A strongly hydrophilic V-shaped groove is employed on the platinum substrate, with a contact angle of approximately 0° determined by the Pt–Ar Lennard–Jones parameters [53]. Van Stralen and Cole pointed out that groove structures generally influence boiling behavior by providing potential vapor embryo retention sites and increasing the effective heat transfer area [65]. Both surface wettability and groove geometry influence bubble nucleation: the hydrophilic groove enhances energy transfer to adjacent liquid atoms, enabling rapid nucleation, while the nanostructured groove, such as a V-shaped groove, increases the effective heat-transfer area compared to a smooth surface [52,53]. This combination promotes localized, reproducible nucleation and improves the statistical stability of the process.

### 2.3. Pressure-Control Plate and Fluid Flow Setup

**Figure 2:**
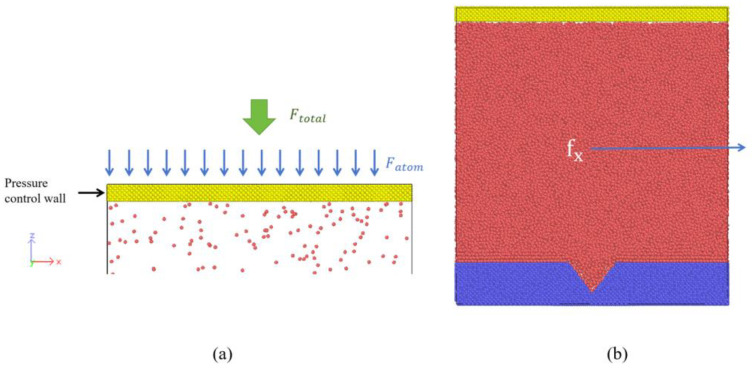
(**a**) Schematic illustration of the pressure-control plate setup; (**b**) Schematic diagram of the fluid flow setup.

In MD simulations with free liquid surfaces, a fixed top wall prevents atom escape but restricts volume expansion and causes pressure buildup [66,67]. To control the system pressure, our study introduces a movable piston-like plate at the top, called the pressure-control plate [40]. The plate moves freely along the *z* direction, driven by the balance between an external force and internal pressure. A total force $F_{total}=PA$ is applied downwards on the plate, shown as Figure 2a, where P is the target pressure and A is the area of the pressure-control plate. This total force is uniformly distributed over all Pt atoms in the plate, so that each atom experiences a constant external force of

(4) $$F_{atom}=\frac{F_{total}}{N_{{Pt}_{controlP}}}=\frac{PA}{N_{{Pt}_{controlP}}},$$

where $N_{{Pt}_{controlP}}$ is the number of atoms in the pressure-control plate.

To investigate bubble nucleation and growth in a flowing liquid film on a solid surface, liquid flow is introduced after the system reaches an equilibrium state. A constant external force is applied on all liquid argon atoms along the positive x direction to drive the film flow, as schematically illustrated in Figure 2b. Each Ar atom is subjected to a uniform force $f_{x}$, whose values range from $4.0\times 10^{-7}\;eV/Å$, to $1.4\times 10^{-6}\;eV/Å$ by a step of $2.0\times 10^{-7}\;eV/Å$.

### 2.4. Ensemble Selection and Simulation Procedure

**Figure 3:**
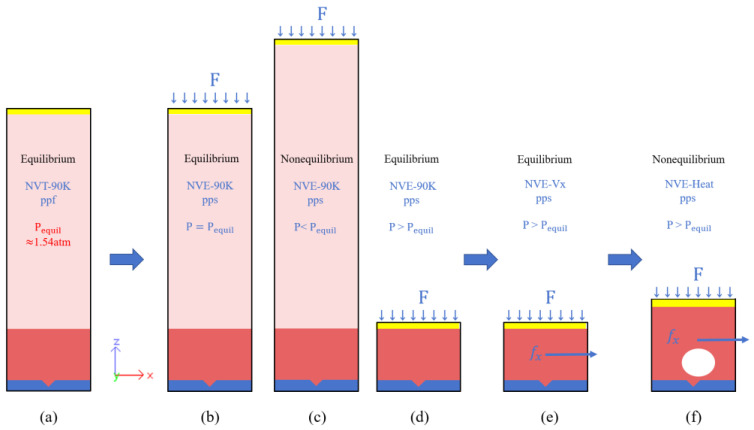
Schematic of the simulation procedure: (**a**) Equilibrated state of the NVT ensemble at 90 K, with no pressure applied to the pressure-control plate; $P_{equil}$ denotes the system equilibrium pressure. (**b**) Equilibrated state of the NVE ensemble at 90 K with the pressure-control plate set to P = $P_{equil}$. (**c**) NVE ensemble at 90 K when P < $P_{equil}$. (**d**) Equilibrated state of the NVE ensemble at 90 K with P > $P_{equil}$. (**e**) Flow establishment stage of the NVE ensemble with the applied body force $f_{x}$; (**f**) Nonequilibrium heating stage. Notes: Black text describes system states; blue text indicates simulation settings; red text labels key outcomes. ‘ppf’ denotes periodic boundaries in x and y directions with fixed z-boundary; ‘pps’ represents periodic x and y boundaries with shrink-wrapped z-boundary.

As illustrated in Figure 3, the entire simulation procedure can be divided into four stages: NVT equilibration without flow (stage 1), NVE pressure equilibration without flow (stage 2), NVE flow establishment (stage 3), and NVE nonequilibrium heating (stage 4).

**Figure 4:**
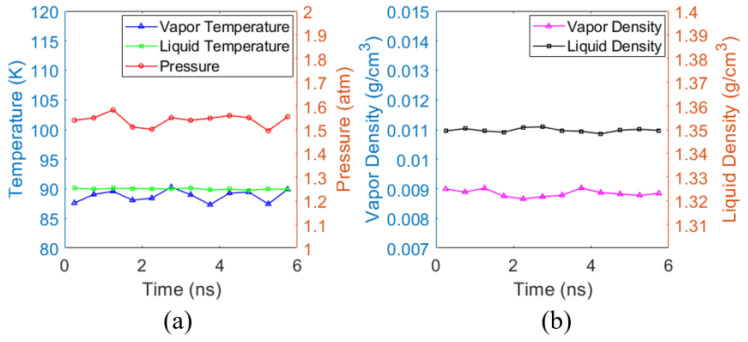
Thermodynamic properties during the final 6 ns of the NVT equilibration stage. (**a**) Liquid temperature, vapor temperature and system pressure. (**b**) Liquid density and vapor density.

The first stage, illustrated in Figure 3a, involves equilibrating the system under the NVT ensemble at 90 K for 27 ns without applying pressure to the top plate. The equilibrium vapor pressure $P_{equil}$ of the system at 90 K is determined to be approximately 1.54 atm (see Figure 4 for NVT equilibrium stage).

In the second stage, the system evolves under the NVE ensemble for 5 ns, with the substrate heat-source layer maintained at 90 K via a Langevin thermostat. The z-direction boundary is set to shrink-wrapped, and a constant target pressure P is applied to the pressure-control plate. When P = $P_{equil}$, the plate remains stationary, confirming the equilibrium vapor pressure of 1.54 atm (Figure 3b). For P < $P_{equil}$, the plate rises due to liquid evaporation and system expansion (Figure 3c), while P > $P_{equil}$ causes the plate to compress the system, suppressing evaporation and establishing stable pressure for subsequent heating (Figure 3d).

To ensure stable and reproducible simulations, all target pressures in this work are set above $P_{equil}$. After NVE equilibration at 90 K under the prescribed target pressure, liquid flow is introduced by applying a constant external body force to all liquid argon atoms along the positive x direction (Figure 3e). Under the applied force and momentum exchange with the top and bottom Pt walls, the liquid gradually develops a steady velocity profile within the channel.

Once the steady flow field has been established, the system proceeds to the fourth stage, as illustrated in Figure 3f. This stage employs the NVE ensemble, with simulation times of 5 ns to capture the dynamics of bubble nucleation and growth. The Pt substrate heat-source layers are instantaneously raised from 90 K to the target temperature, while the pressure-control plate maintains the set pressure. Continuous energy transfer from the heated substrate rapidly increases the local liquid temperature near the substrate, triggering bubble nucleation.

The Velocity-Verlet algorithm [68] is used with a time step of 0.005 ps, and data are recorded every 1000 steps. In this study, all simulations utilize the Large-scale Atomic/Molecular Massively Parallel Simulator (LAMMPS) developed by Plimpton [28], and atom trajectories are visualized using OVITO (Version 3.12.0) [39], an open-source visualization tool developed by Stukowski at Sandia National Laboratory. All simulations were performed using LAMMPS (Version 2 Aug 2023) in a parallel computing environment. Each system contained 87,587 atoms. The calculations were conducted on a 128-core CPU platform with 384 GB of total memory, and a typical simulation case required approximately 2.5 h of wall-clock time.

## 3. Validation of Equilibrium State

### 3.1. Equilibrium State of System Under NVT Ensemble at 90 K

To verify that the system has reached equilibrium under the NVT ensemble at 90 K, the thermodynamic properties during the final 6 ns of the NVT equilibration stage were analyzed, as shown in Figure 4a, which presents the liquid temperature, vapor temperature, and system pressure. The system vapor pressure stabilized at 1.54 atm, in agreement with the vapor pressure of argon, 1.41 atm at 90 K, reported in the literature [69]. Figure 4b shows the corresponding densities of the liquid and vapor phases. The average liquid density is 1.349 g/cm^3^, which is close to the reference value of 1.379 g/cm^3^ from the NIST database [70]. The average vapor density is 0.0088 g/cm^3^, which also agrees well with the NIST value of 0.0074 g/cm^3^ [70].

### 3.2. Equilibrium State of System with Forced Flow Under NVE Ensemble at 90 K

**Figure 5:**
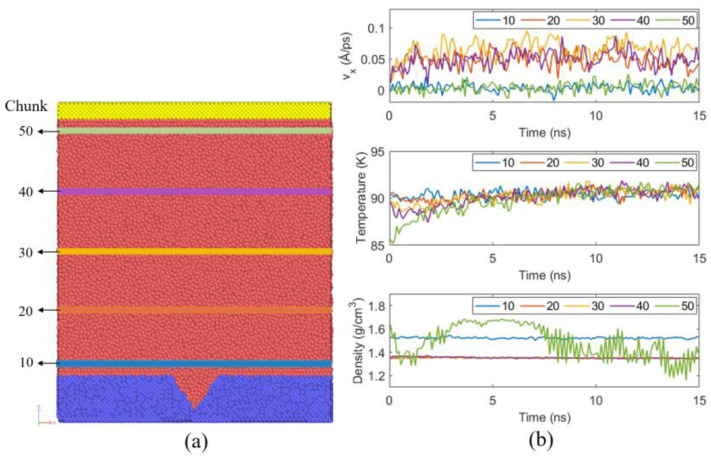
(**a**) Five selected rectangular chunks (chunk numbers 10, 20, 30, 40, 50) along the *z*-direction. (**b**) Time evolution of thermodynamic properties at equilibrium for the case of 10 atm with $f_{x}\;$ = $\;8\times 10^{-7}\;eV/Å$.

A representative case at 10 atm with $f_{x}={8.0\times 10}^{-7}\;eV/Å$, is used to verify steady flow during the third stage under the NVE ensemble. The domain is divided into rectangular chunks along z, and five representative chunks (10, 20, 30, 40, 50) covering the near-substrate, liquid body, and near-plate regions are analyzed, as shown in Figure 5a. In this case, the system is run for 15 ns, and the time evolution of the velocity $v_{x}$, temperature, and density within each chunk are analyzed, as shown in Figure 5b. After approximately 5 ns, all quantities fluctuate around stable mean values without systematic drift, indicating a statistically steady flow.

The relatively higher density in chunk 10 compared with the bulk arises from argon adsorption on the hydrophilic Pt substrate, forming a solid-like layering structure [16,53]. In contrast, larger density fluctuations in chunk 50 result from vertical oscillations of the nearby pressure-control plate. Chunks 20, 30, and 40 thus best represent the bulk liquid density.

**Figure 6:**
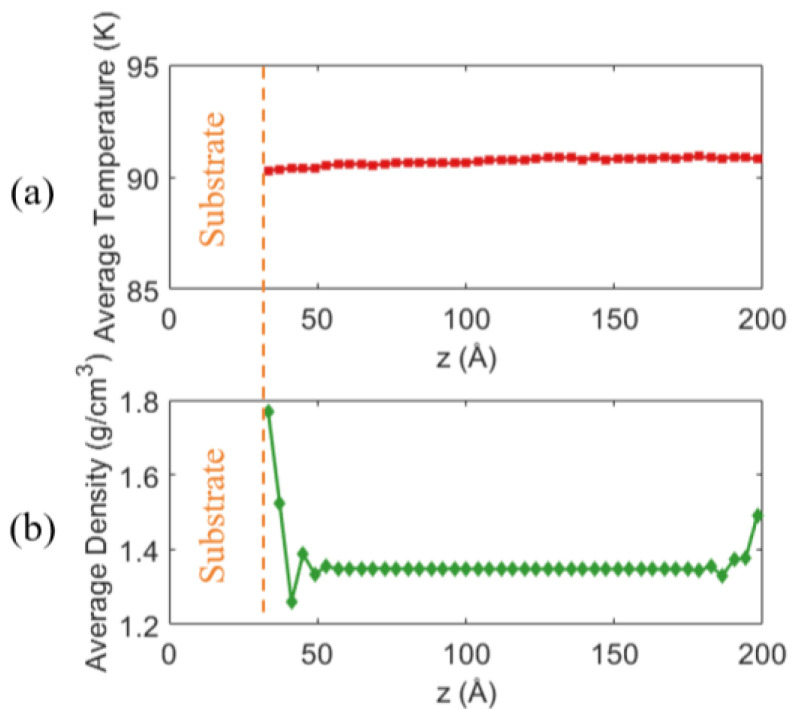
Spatial distributions along the z-direction for the case of 10 atm with $f_{x}={8.0\times 10}^{-7}\;eV/Å$ (data averaged over the final 5 ns of equilibration): (**a**) Temperature distribution; (**b**) Density distribution.

After confirming that the system has reached a steady state, data from the final 5 ns of the simulation are used for time averaging to obtain representative equilibrium values of the physical properties of liquid argon. The resulting z-direction profiles of temperature and density are shown in Figure 6. Each data point corresponds to one chunk.

Figure 6a presents the average Ar temperature distribution along the *z* direction. The temperature within the liquid region is relatively uniform, and the average liquid temperature is 90.7 K, in good agreement with the aimed temperature of 90 K. This indicates that, within the current simulation timescale, the energy distribution of the system has fully relaxed and the temperature control is stable. As shown in Figure 6b, the Ar density exhibits pronounced spatial non-uniformity along the *z* direction. Overall, the average liquid density is 1.35 g/cm^3^, in agreement with the NIST reference [70] value of 1.379 g/cm^3^, confirming the reliability of the model.

In summary, the temperature and density distributions shown in Figure 6 indicate that, for the case of 10 atm with $f_{x}={8.0\times 10}^{-7}\;eV/Å$, the system has established a stable and reasonable flow structure and thermodynamic state during the equilibration stage. This provides a reliable basis for the subsequent statistical analysis and discussion of nucleation behavior.

## 4. Results and Discussion

### 4.1. Flow Velocity Profiles at Steady State Under Different Applied Forces

**Figure 7:**
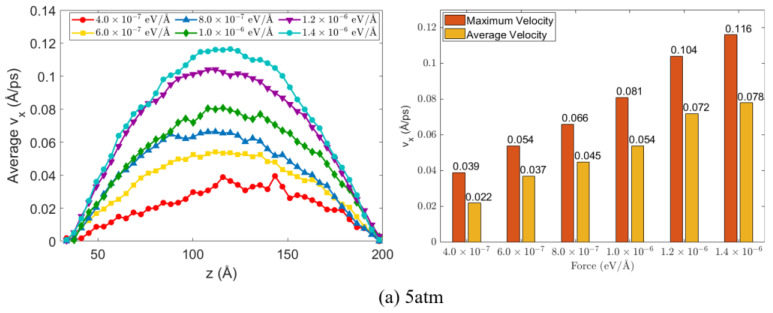

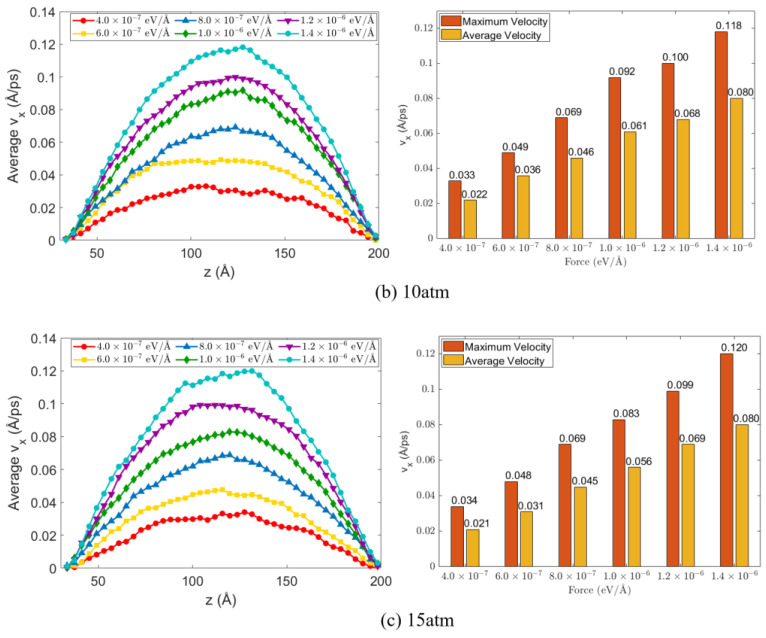
Liquid velocity distribution with different applied body force, along with statistics of maximum and average velocities at equilibrium under (**a**) 5 atm, (**b**) 10 atm, (**c**) 15 atm.

In this work, various body forces from $4.0\times 10^{-7}\;eV/Å$, to $1.4\times 10^{-6}\;eV/Å$ were applied to the Ar atoms, and the resulting steady-state velocity distributions in the liquid film were analyzed, as shown in Figure 7.

The left panels of Figure 7a–c show the velocity profiles of the liquid phase along the *z* direction at 90 K under three system pressures (5 atm, 10 atm, and 15 atm) for different applied forces. In all cases, the velocity distributions are symmetric and parabolic, with maximum velocity at the channel center and near-zero velocity at the walls. The maximum and average velocities, summarized in the right panels, increase linearly with applied force, and the average-to-maximum velocity ratio remains close to 2/3, consistent with confined-channel theory [71]. At a given applied force, both the maximum and average velocities vary slightly with pressure, indicating that the flow velocity is not sensitive to pressure changes and that the applied force provides a stable, well-controlled measure of flow intensity.

### 4.2. Bubble Nucleation and Growth Behavior

**Figure 8:**
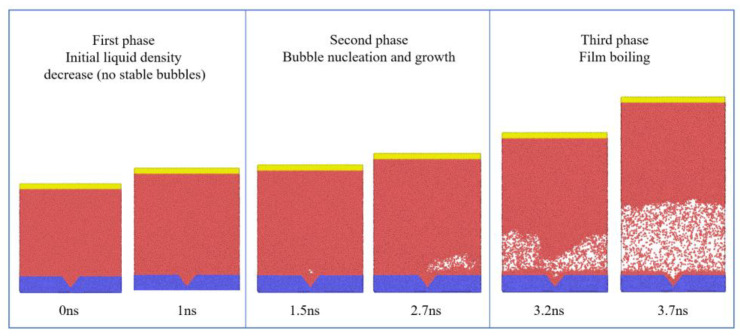
Snapshots of the phase change process in the x-z plane under conditions of 10 atm system pressure, 150 K substrate temperature, and $f_{x\;}$ = $\;1.2\times 10^{-6}\;$eV/Å.

Taking the case with a system pressure of 10 atm, a substrate temperature of 150 K, and an applied force of $f_{x}$= $1.2\times 10^{-6}\;eV/Å$ on Ar atoms as an example, the heating stage can be divided into three phases, as illustrated in Figure 8.

In the first phase, the Pt substrate temperature is instantaneously raised from 90 K to 150 K, causing the adjacent liquid to expand and density to decrease, with no stable bubbles formed. As heating continues, localized overheated regions near the substrate give rise to stabilized vapor nuclei, marking the onset of the second phase: bubble nucleation and growth. During growth, the formed bubbles are affected by flow-induced shear, leading to asymmetric features in both interfacial morphology and spatial distribution. When the bubble continues to grow and completely covers the substrate, the system enters the third phase, in which a vapor film forms. The overall heat transfer efficiency is significantly reduced, exhibiting the features of film boiling.

The focus of this study is on the bubble nucleation and growth phase. A nucleation event is defined as follows: during the simulation, when the volume of a cavity first exceeds the critical nucleation volume and continues to grow thereafter, it is identified as a nucleation event. The time at which nucleation occurs is defined as the nucleation time, with the corresponding volume taken as the critical nucleation volume. Bubble volumes are calculated using a three-dimensional grid-based method with local atomic density [72,73]. The liquid region is divided into cubic cells of 0.2 nm, and a neighborhood radius of 1.2$\sigma_{Ar}$ is defined to check for the presence of argon atoms and $\sigma_{Ar}$ equals 0.34 nm; if no atoms are detected within this range, the cell is marked as a cavity point. Contiguous cavity cells form a bubble, and the largest bubble within the region specified as x from 0 nm to 25.1 nm, y from 0 nm to 4.3 nm, and z from 0 nm to 24.5 nm, is tracked to determine the critical nucleation volume and nucleation time. Thus, the critical nucleation volume [39] is defined as the minimum volume required for a bubble to exist stably and continue to grow and to evolve into a larger bubble rather than to collapse. Cavities smaller than this volume tend to collapse due to their inability to overcome the nucleation barrier, while those exceeding it grow rapidly. This definition not only reflects the transition from unstable cavities to self-sustained bubble growth, but also ensures a unified criterion for comparing nucleation behavior under different thermodynamic and flow conditions.

**Figure 9:**
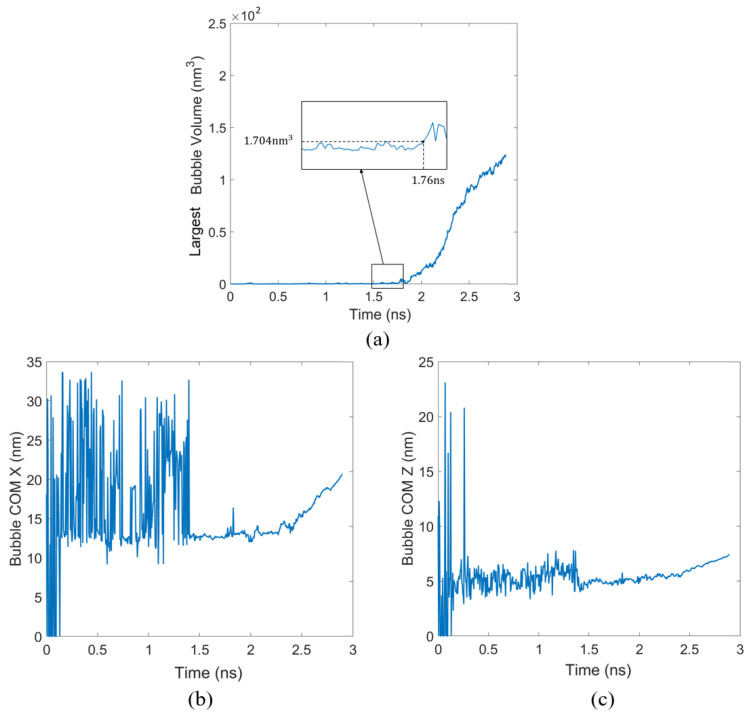
Bubble evolution characteristics under conditions of 10 atm system pressure, 150 K substrate temperature, and $f_{x}\;$ = $\;1.2\times 10^{-6}\;$eV/Å. Time evolution of the (**a**) largest bubble volume and displacement of the bubble’s center of mass (COM) along the (**b**) *x*-direction and (**c**) *z*-direction relative to its initial nucleation position.

In this study, the critical nucleation volume is operationally recognized as the maximum bubble volume reached just before the bubble enters the continuous growth stage, around 1.76 ns in the case of Figure 9a; the volume in the continuous growth stage is always larger than this value. The moment when the bubble volume first surpasses this value is taken as the nucleation time. As shown in Figure 9a, the evolution curve of the largest bubble volume within the bubble tracking region indicates a critical nucleation volume of 1.704 ${nm}^{3}$.

The evolution of the bubble’s center of mass (COM) along x and z was tracked to characterize migration in the flowing liquid. Figure 9b,c shows the time-dependent displacement of the COM relative to the nucleation moment. By combining the bubble volume evolution curves with the centroid migration trajectories, the nucleation time and the critical nucleation volume can be identified, allowing an analysis of the effects of liquid flow on the bubble growth rate and migration behavior.

### 4.3. Effect of Forced Flow on Bubble Nucleation Under Different Operating Conditions

The effect of forced flow on bubble nucleation and growth was investigated under different thermodynamic conditions. Simulations were performed at 150 K with system pressures of 5 atm, 10 atm, and 15 atm, and at 10 atm with temperatures of 145, 150, and 155 K. Liquid flow was driven by applying force $f_{x}$ with values of $4.0\times 10^{-7}\;eV/Å$, $6.0\times 10^{-7}\;eV/Å$, $8.0\times 10^{-7}\;eV/Å$, $1.0\times 10^{-6}\;eV/Å$, $1.2\times 10^{-6}\;eV/Å$ and $1.4\times 10^{-6}\;eV/Å$. To determine the required number of repeated simulations, the statistical stability of nucleation time was examined. Previous studies [52,72,74] show that at least three independent simulations are needed to obtain reliable results. More repetitions improve accuracy: with three to four runs, the average nucleation time differs by about 5.2%, and with four to five runs, it decreases to 1.3%. In this study, we performed five independent simulations for each condition to ensure that the nucleation time and critical bubble size are stable and reproducible. These details have been added to Section 4.3 of the revised manuscript.

**Figure 10:**
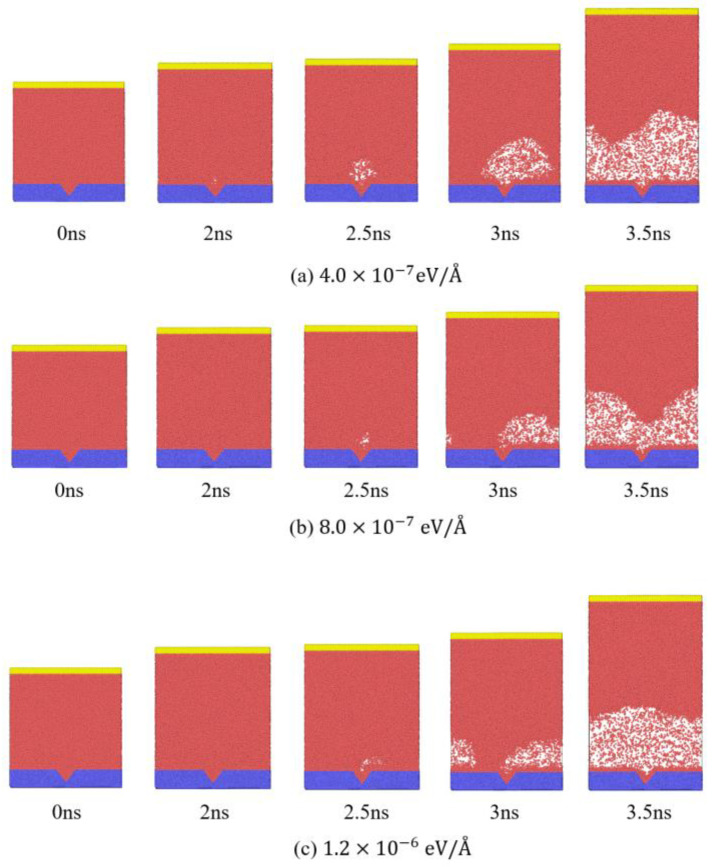
Snapshots of phase evolution at 10 atm system pressure and 150 K substrate temperature for different applied body forces: (**a**) $f_{x}=4.0\times 10^{-7}\;eV/Å$, (**b**) $f_{x}=8.0\times 10^{-7}\;eV/Å$ and (**c**) $f_{x}=1.2\times 10^{-6}\;eV/Å$.

Figure 10 presents the snapshots of phase evolution at a system pressure of 10 atm and a substrate temperature of 150 K for three different values of $f_{x}\;$=$\;4.0\times 10^{-7}\;eV/Å$, $8.0\times 10^{-7}\;eV/Å$ and $1.2\times 10^{-6}\;eV/Å$. When $f_{x}\;$=$\;4.0\times 10^{-7}\;eV/Å$ at 2.5 ns the bubble centroid expands mainly along the *z* direction, with no obvious displacement in the x direction. For $f_{x}\;$=$\;8.0\times 10^{-7}\;eV/Å$, the bubble centroid shows a clear shift toward the positive x direction at 2.5 ns, and the bubble shape becomes asymmetric. When $f_{x}\;$=$\;1.2\times 10^{-6}\;eV/Å$, the displacement of the bubble centroid toward the positive x direction at 2.5 ns becomes more pronounced. At 3.0 ns, the extent of the bubble centroid displacement along the x direction increases with increasing $f_{x}$. These observations indicate that $f_{x}$ is a key factor governing the horizontal displacement of the bubble.

#### 4.3.1. Effect of Forced Flow on Bubble Nucleation Under Different System Pressures

**Figure 11:**
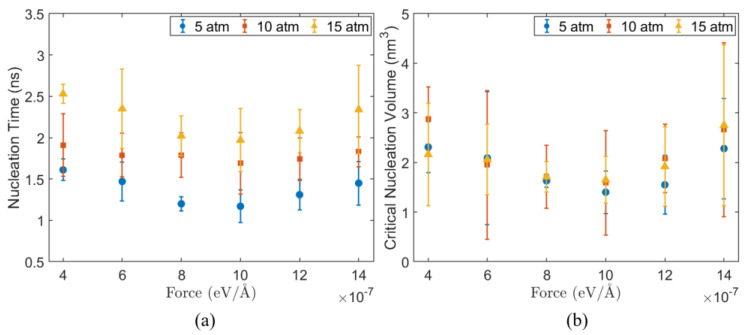
Bubble nucleation characteristics at 150 K substrate temperature under different system pressures and applied body forces: (**a**) nucleation time and (**b**) critical nucleation volume.

At 150 K, the bubble nucleation time and critical nucleation volume of the system under different pressures and applied forces $f_{x}$ are shown in Figure 11. First, the nucleation time increases with increasing pressure for all $f_{x}$, as shown in Figure 11a. For example, at $f_{x}$ = $4.0\times 10^{-7}\;eV/Å$, the nucleation time increases from 1.61 ns at 5 atm to 1.91 ns at 10 atm, and further extends to 2.53 ns at 15 atm, corresponding to an increase of about 57%. Even under the condition with the fastest nucleation ($f_{x}$ = $1.0\times 10^{-6}\;eV/Å$), the nucleation time at 15 atm (1.97 ns) remains higher than that at 5 atm (1.17 ns).

Under the same pressure, the nucleation time exhibits a non-monotonic dependence on $f_{x}$. As $f_{x}$ increases from $4.0\times 10^{-7}\;eV/Å$ to $1.0\times 10^{-6}\;eV/Å$, nucleation time decreases, reaching a minimum, and then rises at $f_{x}\;$=$\;1.4\times 10^{-6}\;eV/Å$. For example, at 5 atm, the nucleation time decreases from 1.61 ns to a minimum value of 1.17 ns, and then increases to 1.45 ns.

The critical nucleation volume exhibits a trend similar to nucleation time, decreasing and then increasing with $f_{x}$, and reaching a minimum near the $f_{x}$ corresponding to the shortest nucleation time (Figure 11b). At 5 atm, it decreases from 2.31 ${nm}^{3}$ at $f_{x}\;$=$\;4.0\times 10^{-7}\;eV/Å$ to 1.40 ${nm}^{3}$ at $f_{x}\;$=$\;1.0\times 10^{-6}\;eV/Å$, then increases to 2.28 ${nm}^{3}$ at $f_{x}$ = $1.4\times 10^{-6}\;eV/Å$. Under the same applied driving condition, the critical nucleation volume shows only weak dependence on pressure. Previous work [75] reported a critical nucleation radius of approximately 2.0 nm, corresponding to a critical volume on the order of 1–10 nm^3^. The critical nucleation volumes obtained in Figure 11 fall within the same order of magnitude. This agreement in scale supports the physical consistency of the nucleation identification approach used.

**Figure 12:**
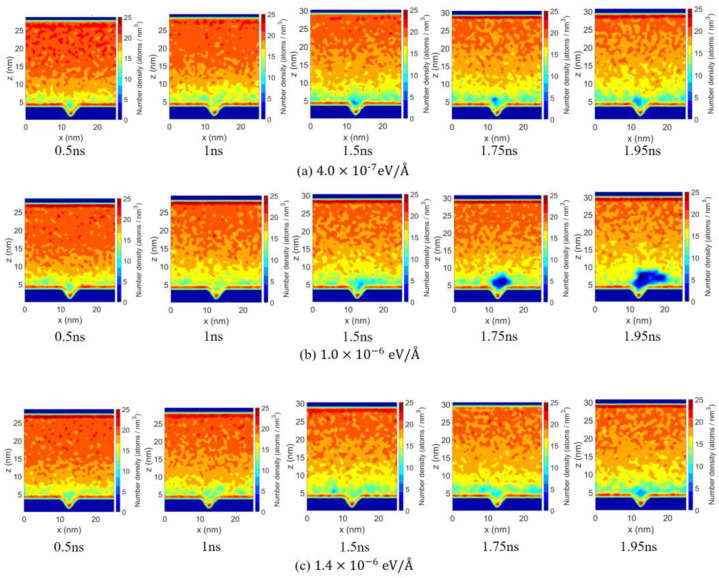
Argon number density distributions (x-z plane) at different times for three applied body forces under 10 atm and 150 K: (**a**) $f_{x}=4.0\times 10^{-7}\;eV/Å$, (**b**) $f_{x}=1.0\times 10^{-6}\;eV/Å$ and (**c**) $f_{x}=1.4\times 10^{-6}\;eV/Å$.

From the perspective of pressure effects, the nucleation time increases with increasing pressure for all $f_{x}$. A higher system pressure increases the volume work that need to be overcome during the bubble formation, which thermodynamically raises the nucleation free-energy barrier and consequently leads to a longer nucleation time. The effect of $f_{x}$ is non-monotonic: low-density regions near the substrate appear earlier for $f_{x}\;$=$\;1.0\times 10^{-6}\;eV/Å$ than for $f_{x}$ = $4.0\times 10^{-7}\;eV/Å$ (Figure 12). This indicates that the applied body force enhances local overheating and density fluctuations, enabling the system to reach critical nucleation conditions more rapidly and shortening nucleation time.

When $f_{x}$ increases to $1.4\times 10^{-6}\;eV/Å$, the flow changes from promoting to suppressing nucleation. After 1.5 ns, compared with system with $f_{x}=1\times 10^{-6}\;eV/Å$, as shown in Figure 12b, although a low-density region in Figure 12c ($f_{x}=1.4\times 10^{-6}\;eV/Å$) can still be observed near the substrate, their shape and location become unstable. Under higher $f_{x}$ ($f_{x}=1.4\times 10^{-6}\;eV/Å$), the strong flow perturbs the bubble–liquid interface, disrupting small nuclei before they stabilize. Under such conditions, only when a bubble nucleus grows to a larger volume can the bulk effects inside the bubble and the contribution of interfacial energy overcome the externally imposed shear disturbances, resulting in a higher critical nucleation volume and longer nucleation time.

Pressure and the applied force $f_{x}$, play distinct but interrelated roles in bubble nucleation. With increasing pressure, the nucleation time shows an overall increasing trend, indicating that pressure imposes a global constraint on nucleation by raising the thermodynamic energy barrier that must be overcome. $f_{x}$ mainly regulates the rate at which the system crosses this barrier through kinetic processes.

#### 4.3.2. Effect of Forced Flow on Bubble Nucleation Under Different Substrate Temperatures

**Figure 13:**
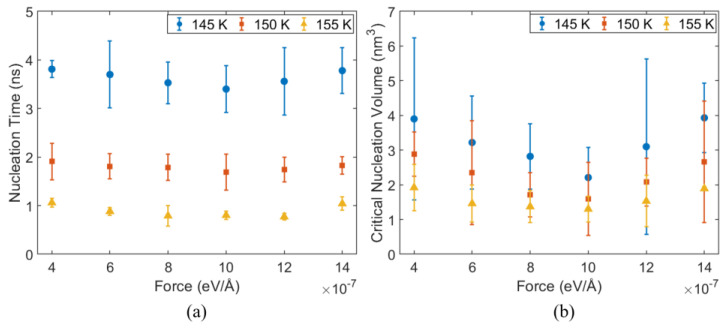
Bubble nucleation characteristics at 10 atm system pressure under different substrate temperatures and applied body forces: (**a**) nucleation time and (**b**) critical nucleation volume.

At a system pressure of 10 atm, the nucleation time and critical nucleation volume under different temperatures (145 K, 150 K, and 155 K) and different applied forces $f_{x}$ are shown in Figure 13.

As shown in Figure 13a, under the same applied force, the nucleation time decreases significantly with increasing temperature. Taking $f_{x}\;$=$\;1.0\times 10^{-6}\;eV/Å$ as an example, the nucleation times at 145 K, 150 K, and 155 K are 3.40 ns, 1.69 ns, and 0.80 ns, respectively. Increasing the temperature from 145 K to 155 K shortens the nucleation time by about 76%, indicating that temperature plays an important role in governing bubble nucleation. At a fixed temperature, the nucleation time shows a non-monotonic dependence on $f_{x}$, first decreasing and then increasing. At 145 K, the minimum nucleation time of 3.40 ns is reached at $f_{x}\;$=$\;1.0\times 10^{-6}\;eV/Å$.

As shown in Figure 13b, at a fixed temperature, the critical nucleation volume exhibits a non-monotonic dependence on the applied force, consistent with the trend observed for the nucleation time. The critical volume reaches a minimum at an intermediate value of $f_{x}$ and increases again under larger $f_{x}$. At 145 K, the critical volume decreases from 3.90 nm^3^ ($f_{x}\;$=$\;4.0\times 10^{-7}\;eV/Å$) to 2.21 nm^3^ ($f_{x}\;$=$\;1.0\times 10^{-6}\;eV/Å$), and then rises to 3.93 nm^3^ ($f_{x}\;$=$\;1.4\times 10^{-6}\;eV/Å$).

Our analysis shows that, at a fixed system pressure, bubble nucleation behavior is jointly regulated by the substrate temperature and the applied force $f_{x}$, while their roles differ. As shown in Figure 13, over the entire range of $f_{x}$, increasing the substrate temperature leads to a pronounced reduction in nucleation time and an overall decrease in the critical nucleation volume, indicating that temperature is the primary controlling factor determining the ease of bubble nucleation by lowering the nucleation energy barrier at the thermodynamic level. The influence of $f_{x}$ is mainly manifested through kinetic mechanisms that regulate the nucleation pathway. In nanoscale confined systems, a moderate increase in driving force accelerates local momentum and energy transport and enhances nonequilibrium fluctuations near the interface, thereby lowering the effective nucleation barrier and shortening the nucleation time. This trend is consistent with previous molecular dynamics studies at the nanoscale [44,46]. However, when the driving force is further increased, interfacial and confinement effects intrinsic to nanoscale systems begin to dominate. Mechanisms such as hindered liquid replenishment, reduced stability of vapor embryos, and vapor shielding can emerge, which delay or alter the nucleation pathway and lead to non-monotonic behavior [43,45,47]. Therefore, at the nanoscale, flow does not simply promote nucleation; rather, it acts as a dual regulator, governed by the competition between transport enhancement and interfacial destabilization, resulting in both promotion and suppression of nucleation.

### 4.4. Effect of Flowing on Bubble Growth Behavior

**Figure 14:**
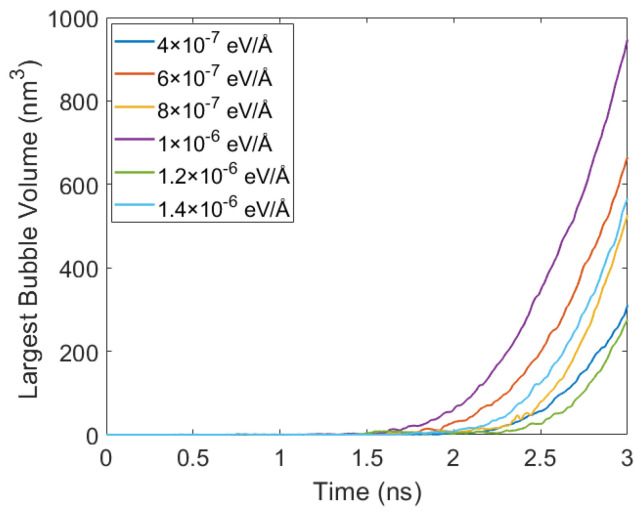
Temporal evolution of the largest bubble volume in liquid argon at 150 K and 10 atm for different applied body forces.

Taking the case of 150 K and 10 atm as an example, Figure 14 shows the time evolution of bubble volume under different $f_{x}$. Initially, no stable bubbles exist, but once a nucleus forms, the bubble volume grows rapidly in a nonlinear manner. The bubble growth rate exhibits a non-monotonic dependence on $f_{x}$, first increasing and then decreasing as $f_{x}$ increases. The slowest growth is observed at $f_{x}\;$=$\;4.0\times 10^{-7}\;eV/Å$. When $f_{x}$ increases to $1.0\times 10^{-6}\;eV/Å$, the bubble volume grows more rapidly. However, when $f_{x}$ is further increased to $1.4\times 10^{-6}\;eV/Å$, the slope of the volume–time curve no longer increases, indicating that bubble growth is no longer further promoted under stronger flow conditions.

**Figure 15:**
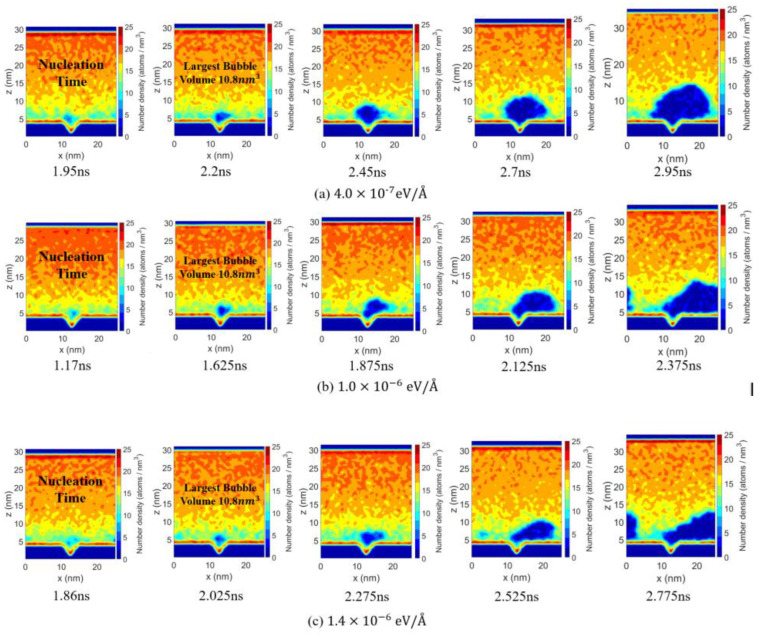
Argon number density distributions (x-z plane) at different times for three applied body forces under 10 atm and 150 K: (**a**) $f_{x}=4.0\times 10^{-7}\;eV/Å$, (**b**) $f_{x}=1.0\times 10^{-6}\;eV/Å$ and (**c**) $f_{x}=1.4\times 10^{-6}\;eV/Å$.

To better analyze bubble growth behavior, the Ar number density distribution snapshots are presented in Figure 15. The first column shows the bubble at nucleation, and the second column marks when the bubble reaches 10.8 ${nm}^{3}$, serving as a reference for initial post-nucleation volume. Subsequent snapshots are taken at 0.25 ns intervals from this point, allowing comparison of bubble growth and interfacial morphology under different $f_{x}$ at the same post-nucleation times.

The number density map in Figure 15a shows that, under the diving force of $f_{x}\;$=$\;4.0\times 10^{-7}\;eV/Å$, the bubble remains near the nucleation region, with growth dominated by local heating-induced evaporation, resulting in a low growth rate. In Figure 15b, under the force of $f_{x}\;$=$\;1.0\times 10^{-6}\;eV/Å$, enhanced migration and elongation along the flow direction strengthen convective heat transport, promoting faster bubble growth.

In Figure 15c, when $f_{x}$ is increased to a larger value ($1.4\times 10^{-6}\;eV/Å$), fluid flow induces stronger interfacial fluctuations of the bubble, and the low-density region becomes more susceptible to shear stretching. The combined effects of strong shear and flow-induced disturbances continuously act on the bubble–liquid interface, hindering continuous expansion and limiting further growth.

**Figure 16:**
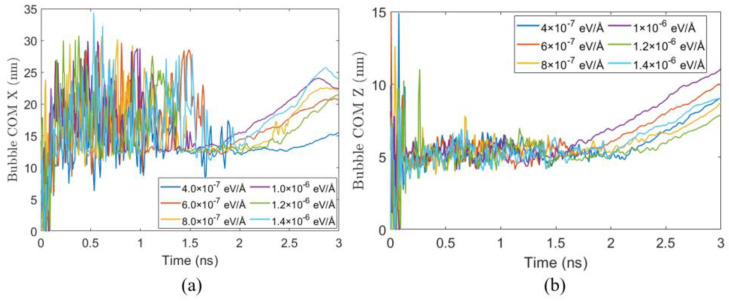
Temporal evolution of displacement of the bubble’s center of mass (COM) along the (**a**) *x*-direction and (**b**) *z*-direction relative to its initial nucleation position.

To further investigate the effect of flow on the spatial evolution of bubble growth, Figure 16 presents the time evolution of the bubble’s center of mass in the *x* and *z* directions under different $f_{x}$. During the early heating stage (approximately 0 ns–0.5 ns), the centroid exhibits large fluctuations due to initial bubble formation, thermal effects, and interfacial oscillations. As the bubble stabilizes and grows, the centroid trajectories under all conditions gradually converge and become smoother.

As shown in Figure 16a, under all operating conditions, the displacement of the bubble centroid along the x direction increases in an approximately linear manner after nucleation, showing that once nucleated, the bubble is continuously transported by the bulk liquid flow. When the driving force increases from $4.0\times 10^{-7}\;eV/Å$ to $1.4\times 10^{-6}\;eV/Å$, the x-direction displacement at 3 ns increases from about 5 Å to more than 30 Å, a six-fold increase, highlighting the strong effect of flow intensity on bubble migration along the x direction.

Figure 16b shows the bubble centroid evolution along z, perpendicular to the substrate. Movement in this direction is mainly due to bubble volume expansion. Even under the largest applied force of $1.4\times 10^{-6}\;eV/Å$, the displacement of the bubble centroid along the *z* direction remains smaller than that along the *x* direction, indicating that flow has a limited effect on bubble migration in the *z* direction.

**Figure 17:**
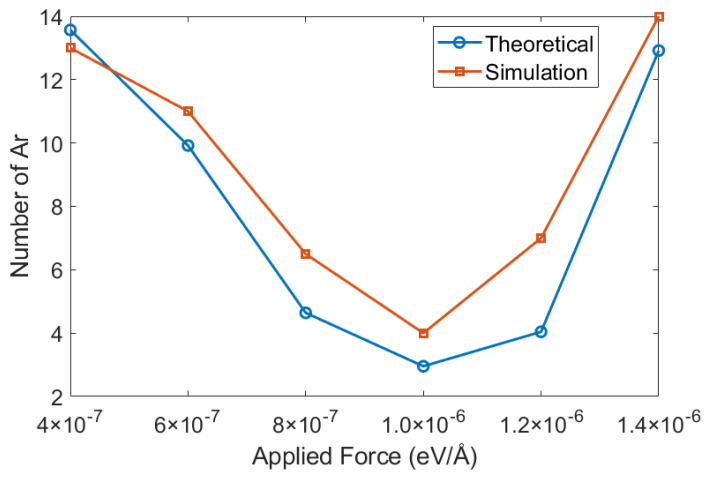
Comparison between simulation and theoretical predictions of gas atom numbers in nucleated bubbles at 5 atm and 150 K under different force-driven flow conditions.

To assess the agreement between theoretical predictions and simulation results, we compared the number of gas atoms in the nucleated bubbles with estimates from the critical radius-based estimation. The bubble volumes extracted from simulations were converted into equivalent critical radii, from which nucleation temperatures and expected molecule numbers were calculated using the ideal-gas relation under the imposed system pressure. The actual number of gas atoms in the bubble was directly counted from the simulation trajectories. Figure 17 shows this comparison at 5 atm and 150 K for different flow forces.

Overall, the simulation values fall within the same order of magnitude as the theoretical estimates and follow consistent trends. This agreement indicates that the critical radius-based estimation not only provides a quantitative reference but also validates the reliability of the molecular simulation results for bubble nucleation volumes under the studied conditions.

## 5. Conclusions

In this work, molecular dynamics simulations were performed to investigate bubble nucleation and growth in a force-driven liquid film under controlled pressure. A stable Poiseuille flow was generated by applying a body force ($4.0\times 10^{-7}\;eV/Å$ to $1.4\times 10^{-6}\;eV/Å$) to liquid argon atoms.

Bubble nucleation is governed primarily by thermodynamic conditions, with temperature and pressure determining the nucleation barrier. Under fixed thermodynamic conditions, the applied force influences nucleation through kinetic mechanisms and exhibits a non-monotonic effect. As the applied body force increases from $4.0\times 10^{-7}\;eV/Å$ to $1.4\times 10^{-6}\;eV/Å$, moderate forcing accelerates nucleation by enhancing near-wall heat transfer and density fluctuations, whereas excessive forcing suppresses nucleation due to strong shear and interfacial disturbances that destabilize incipient nuclei.

During bubble growth, increasing the applied force from $4.0\times 10^{-7}\;eV/Å$ to $1.0\times 10^{-6}\;eV/Å$ promotes growth by enhancing convective heat transfer and facilitating elongation and migration along the flow direction. When the applied force is further increased to $1.4\times 10^{-6}\;eV/Å$, the growth rate no longer increases due to strong shear and interfacial fluctuations that destabilize the bubble–liquid interface. Flow mainly drives migration in the x direction, while displacement in the *z* direction remains much weaker throughout the force range from $4.0\times 10^{-7}\;eV/Å$ to $1.4\times 10^{-6}\;eV/Å$.

In summary, the molecular dynamics results reveal the coupled thermodynamic and kinetic roles of pressure, temperature, and force-driven flow in governing bubble nucleation, growth, and migration. The findings clarify how flow modifies nucleation pathways and growth dynamics under controlled-pressure conditions, and provide molecular-level insights for regulating phase-change behavior in confined liquid film and at solid–liquid interfaces, with implications for the design of advanced thermal surfaces.

## Figures and Tables

**Table 1:** Lennard-Jones parameters [53].

Interaction Type	ε/eV	σ/nm
Ar-Ar	0.0104	0.3405
Pt-Pt	0.5219	0.2475
$Ar-{Pt}_{substrate}$	0.0737	0.2940
$Ar-{Pt}_{controlP}$	0.0737	0.2940

## Data Availability

The raw data supporting the conclusions of this article will be made available by the authors on request.

## Abbreviations

The following abbreviations are used in this manuscript:

CNT	Classical nucleation theory
COM	Center of mass
FCC	Face-centered cubic
LAMMPS	Large-scale Atomic/Molecular Massively Parallel Simulator
MD	Molecular dynamics
NIST	National Institute of Standards and Technology
NVE	Microcanonical ensemble
NVT	Canonical ensemble
OVITO	Open Visualization Tool
